# A Genome-Centric Approach Reveals a Novel Glycosyltransferase from the GA A07 Strain of *Bacillus thuringiensis* Responsible for Catalyzing 15-*O*-Glycosylation of Ganoderic Acid A

**DOI:** 10.3390/ijms20205192

**Published:** 2019-10-20

**Authors:** Te-Sheng Chang, Tzi-Yuan Wang, Tzu-Yu Hsueh, Yu-Wen Lee, Hsin-Mei Chuang, Wen-Xuan Cai, Jiumn-Yih Wu, Chien-Min Chiang, Yu-Wei Wu

**Affiliations:** 1 Department of Biological Sciences and Technology, National University of Tainan, Tainan 70005, Taiwan; mozyme2001@gmail.com (T.-S.C.); vuvu99983@gmail.com (T.-Y.H.); s10458017@gm2.nutn.edu.tw (Y.-W.L.); tiffany170420@gmail.com (H.-M.C.); amy19990630@yahoo.com.tw (W.-X.C.); 2 Biodiversity Research Center, Academia Sinica, Taipei 11529, Taiwan; tziyuan@gmail.com; 3 Department of Food Science, National Quemoy University, Kinmen County 892, Taiwan; wujy@nqu.edu.tw; 4 Department of Biotechnology, Chia Nan University of Pharmacy and Science, No. 60, Erh-Jen Rd., Sec. 1, Jen-Te District, Tainan 71710, Taiwan; cmchiang@mail.cnu.edu.tw; 5 Graduate Institute of Biomedical Informatics, College of Medical Science and Technology, Taipei Medical University, Taipei 11031, Taiwan; 6 Clinical Big Data Research Center, Taipei Medical University Hospital, Taipei 11031, Taiwan

**Keywords:** Nanopore sequencing, ganoderic acid, *Bacillus thuringiensis*, biotransformation, glycosyltransferase, whole genome sequencing

## Abstract

Strain GA A07 was identified as an intestinal *Bacillus* bacterium of zebrafish, which has high efficiency to biotransform the triterpenoid, ganoderic acid A (GAA), into GAA-15-*O*-β-glucoside. To date, only two known enzymes (BsUGT398 and BsUGT489) of *Bacillus subtilis* ATCC 6633 strain can biotransform GAA. It is thus worthwhile to identify the responsible genes of strain GA A07 by whole genome sequencing. A complete genome of strain GA A07 was successfully assembled. A phylogenomic analysis revealed the species of the GA A07 strain to be *Bacillus thuringiensis*. Forty glycosyltransferase (GT) family genes were identified from the complete genome, among which three genes (*FQZ25_16345*, *FQZ25_19840*, and *FQZ25_19010*) were closely related to BsUGT398 and BsUGT489. Two of the three candidate genes, *FQZ25_16345* and *FQZ25_19010*, were successfully cloned and expressed in a soluble form in *Escherichia coli*, and the corresponding proteins, BtGT_16345 and BtGT_19010, were purified for a biotransformation activity assay. An ultra-performance liquid chromatographic analysis further confirmed that only the purified BtGT_16345 had the key biotransformation activity of catalyzing GAA into GAA-15-*O*-β-glucoside. The suitable conditions for this enzyme activity were pH 7.5, 10 mM of magnesium ions, and 30 °C. In addition, BtGT_16345 showed glycosylation activity toward seven flavonoids (apigenein, quercetein, naringenein, resveratrol, genistein, daidzein, and 8-hydroxydaidzein) and two triterpenoids (GAA and antcin K). A kinetic study showed that the catalytic efficiency (k_cat_/K_M_) of BtGT_16345 was not significantly different compared with either BsUGT398 or BsUGT489. In short, this study identified BtGT_16345 from *B. thuringiensis* GA A07 is the catalytic enzyme responsible for the 15-*O*-glycosylation of GAA and it was also regioselective toward triterpenoid substrates.

## 1. Introduction

Glycosyltransferase (GT, EC 2.4.x.y) exists in all living beings and is able to catalyze the glycosylation of molecules such as proteins, nucleic acids, polysaccharides, and lipids. Most GTs use a nucleotide-activated sugar donor, such as uridine diphosphate (UDP)-glucose, in the catalytic reaction. According to a carbohydrate-activating enzyme (CAZy) database, GTs are classified into 107 families [1]. Among them, GTs that use small molecules (such as flavonoids or triterpenoids) as sugar acceptors are classified into the GT1 family. Many members of the GT1 family with activities toward flavonoids have been identified [2,3], however, very few GT1 family members with glycosylation activities toward triterpenoids were reported until recently [4].

Biotransformation of xenobiotics by either a microorganism’s whole cells or purified enzymes may form more-bioactive metabolites than the precursor molecules [5,6,7,8,9]. Among different biotransformations, glycosylation was shown to improve water solubility, stability, and bioactivities of flavonoids, such as anti-oxidant and anti-allergic activities [10,11,12]. Glycosylation of triterpenoids to form saponins can also improve some bioactivities of the triterpenoid precursors. For examples, dozens of reports showed that triterpenoid glycosides, ginseng saponins, from the medicinal plant ginseng, possess more bioactivities involved in the central nervous system, cardiovascular system, immune system, anticarcinogenic activities, and diabetes mellitus, than do ginseng triterpenoid aglycones [13]. Therefore, using GT to biotransform xenobiotics to new glycoside compounds is a worthy field of study.

Ganoderic acid A (GAA) is a triterpenoid isolated from the medicinal fungus, *Ganoderma lucidum* [14]. In addition to GAA, more than 300 different kinds of triterpenoids have been isolated from *Ganoderma* spp. [15], and studies suggested that these triterpenoids may possess many bioactivities [15,16,17]. Despite numerous kinds of triterpenoids having been identified from *G. lucidum*, very few natural *Ganoderma* triterpenoids exist in the form of glycosides (saponins) [15]. In addition, only five microbial GTs were found to biotransform triterpenoids into new bioactive derivatives [1,2,3,4]. Taken together, finding GTs that target *Ganoderma* triterpenoids could potentially expand the diversities of both GT enzymes and *Ganoderma* triterpenoids.

Our previous study identified an intestinal bacterium of zebrafish, *Bacillus* sp. GA A07 strain, which could biotransform GAA into GAA-15-*O*-β-glucoside [18]. In order to identify the GTs of the GA A07 strain responsible for this triterpenoid biotransformation, the complete genome of the strain was resolved using both Nanopore long-read and BGI short-read sequencing technologies. Candidate GT genes were then discovered by comparing them to the CAZy database [1] and also subsequently searching potential sequences against five triterpenoid-glycosylation genes [4,19,20,21,22,23,24]. These candidate genes were then subcloned and overexpressed in *Escherichia coli*; the biotransformation activities of the purified recombinant GTs were also determined.

## 2. Results

### 2.1. Comparison of GAA-15-O-β-Glucoside Production between B. subtilis ATCC 6633 and Bacillus sp. GA A07

**Figure 1:**
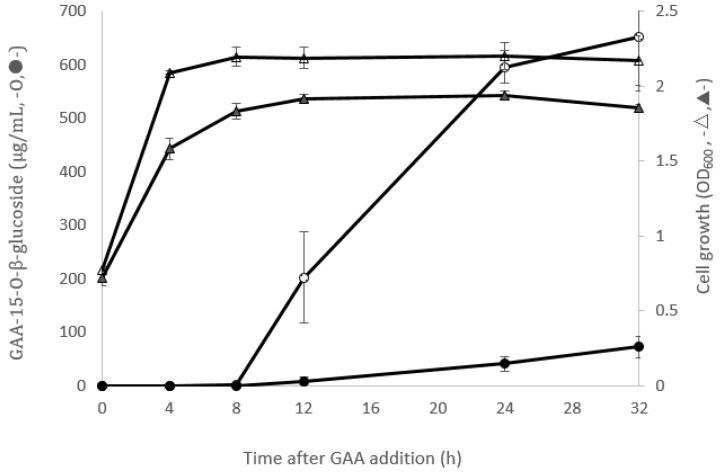
Time course of ganoderic acid A (GAA)-15-*O*-β-glucoside production (circle symbols) and cell growth (triangle symbols) by either *Bacillus subtilis* ATCC 6633 (solid) or *Bacillus sp*. GA A07 (open). The two strains were cultivated in Luria-Bertani (LB) media with shaking at 180 rpm and 30 °C. GAA at 1 mg/mL was added to the fermentation broth as the optical density (OD) at 600 nm of the culture reached 0.6, and cultivation continued for another 32 h. During cultivation, the fermentation broth was analyzed by ultra-performance liquid chromatography (UPLC). The UPLC operating conditions are described in the Materials and Methods section.

Our previous study identified two *Bacillus* strains with the ability to biotransform GAA to GAA-15-*O*-β-glucoside, *B. subtilis* ATCC 6633 [4] and *Bacillus* sp. GA A07 [18]. To compare the biotransformation activity between the two strains, fermentation broths of the two strains fed with GAA were analyzed by ultra-performance liquid chromatography (UPLC) during cultivation. Results showed that *Bacillus* sp. GA A07 possessed 12.5-fold higher GAA biotransformation activity than *B. subtilis* ATCC 6633 after a 24 h incubation of GAA (Figure 1). Based on these results, the complete genome of the GA A07 strain was resolved to identify the GTs responsible for the triterpenoid biotransformation.

### 2.2. Genome Sequencing, Assembly, Annotation, and Reclassification of the GA A07 Strain

**Figure 2:**
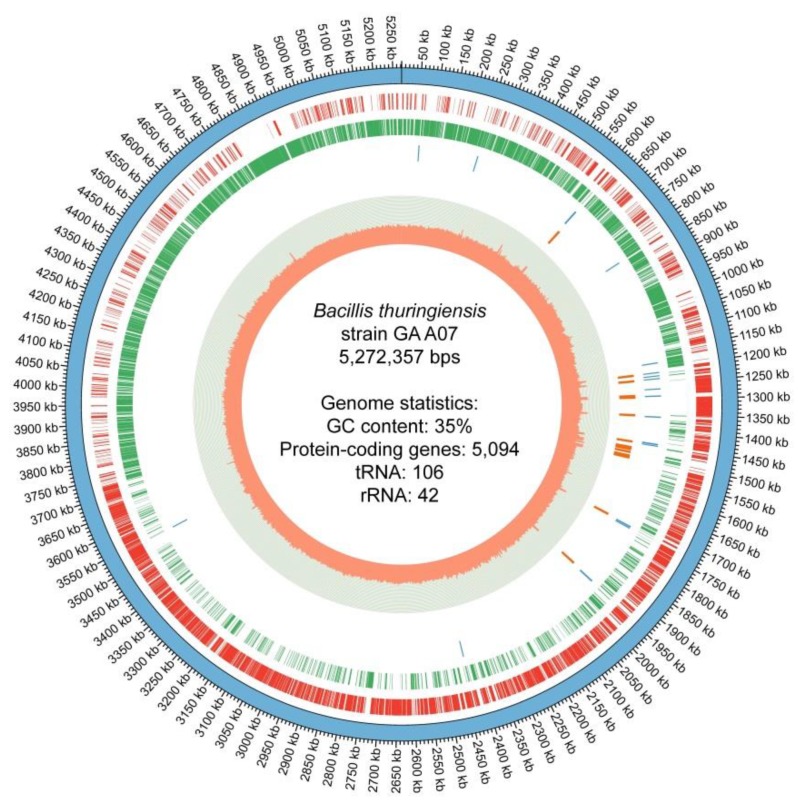
Circular genome map of the *Bacillus thuringiensis* GA A07 strain. The six rings from the outer to inner side represent (1) assembled scaffolds, (2) genes in the forward strand, (3) genes in the reverse-complement strand, (4) transfer RNA (tRNA) genes, (5) ribosomal RNA (rRNA) genes, and (6) the GC content distribution per 1000 bps.

Genome sequencing of *Bacillus* sp. GA A07 was performed in order to determine the enzymes that contribute to GAA glycosylation. Totally 1,217,502,092 base pairs (bps) were sequenced from 142,886 Nanopore reads. The average read length was 8521 bps. The assembly process (outlined in the Materials and Methods section) yielded a complete circular genome along with four circular plasmids (GenBank BioProject accession no. PRJNA557365; Genome accession no. CP042270). The genome size was 5,272,357 bps with a G+C percentage of 35.33%. Totally, 5094 putative protein-coding genes, 106 transfer RNA (tRNA) genes, and 42 ribosomal RNA (rRNA) genes were annotated for the GA A07 strain, as shown in the circular genome map (Figure 2).

**Figure 3:**
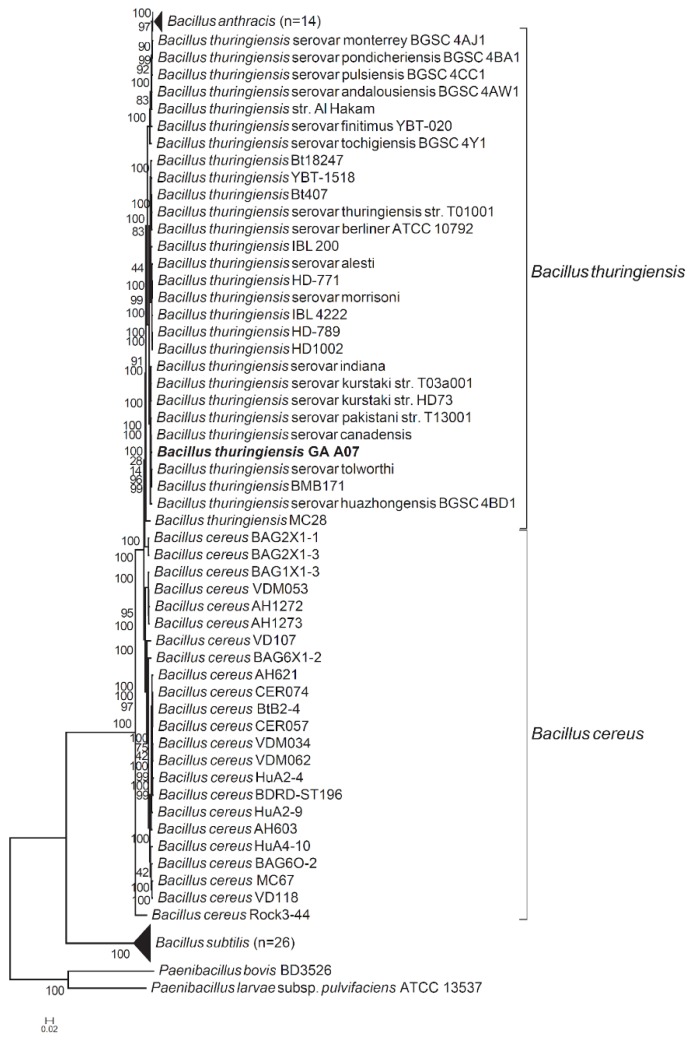
Phylogenetic tree for the *Bacillus thuringiensis* GA A07 strain. The tree was built from 250 single-copy marker genes. Two *Paenibacillus* genomes were included as outgroups. The GA A07 strain is marked in bold font. See the Materials and Methods section for details.

The 16S gene tree was only able to group the GA A07 strain with other *Bacillus* species (including *B. subtilis*, *B. thuringiensis*, *B. cereus*, and *B. anthracis*) [18]; however, the actual species to which it belonged could not be determined using only the 16S rRNA gene. Thus, we employed a two-step method to identify which species the GA A07 strain belongs to. First, we used three different approaches [25,26,27], namely average nucleotide identity (ANI), average amino acid identity (AAI), and a tetra correlation search (TCS), to find the most closely related species to the GA A07 strain. All methods identified *B. thuringiensis* as the closest species (*B. thuringiensis* serovar *canadensis* identified by both the ANI and AAI approaches and *B. thuringiensis* BMB171 identified by the TCS approach). We then built a phylogenetic tree (Figure 3) from 250 single-copy marker genes (the full list of marker genes can be found in Table S1). Figure 3 revealed the GA A07 strain indeed belongs to the group of *B. thuringiensis*.

### 2.3. Phylogenetic Analysis of GTs from the GA A07 Strain

**Figure 4:**
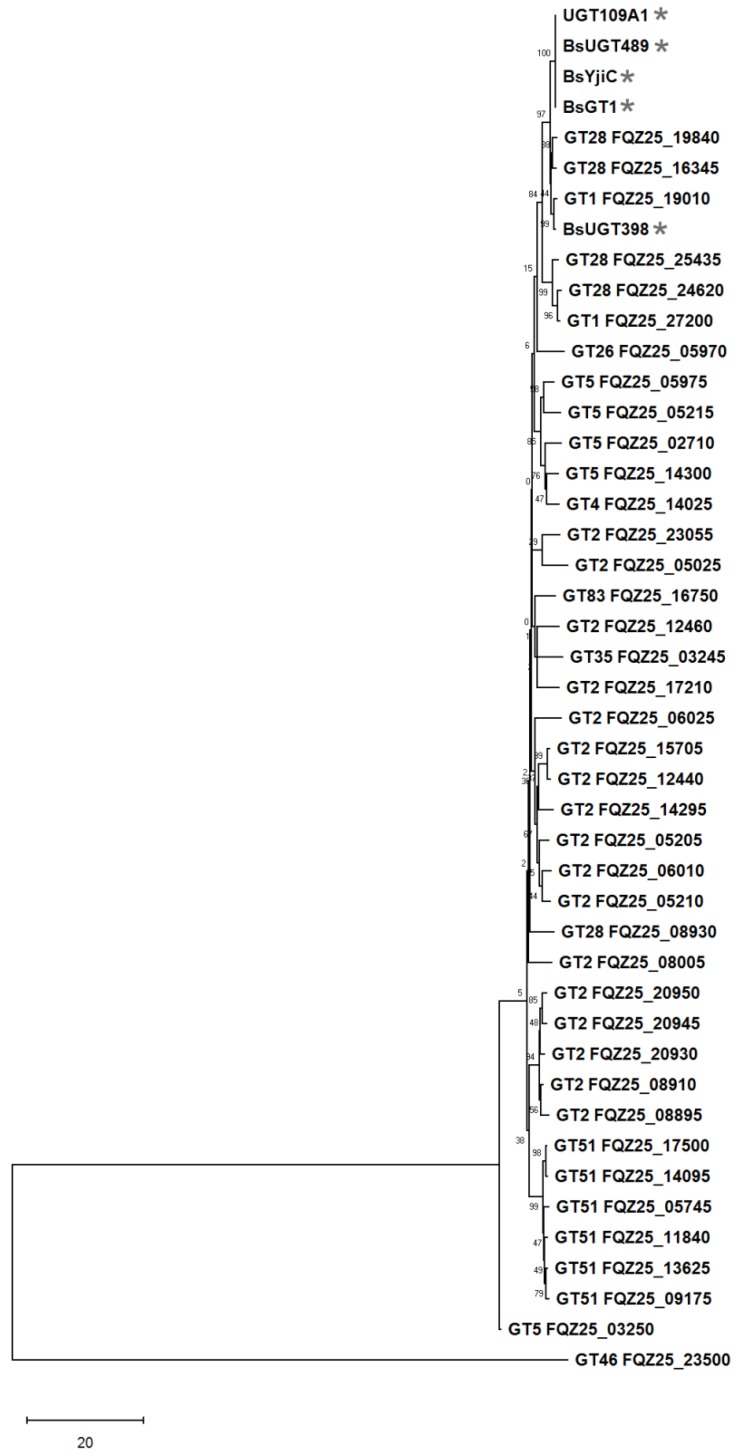
Molecular phylogenetic analysis of glycosyltransferase (GT) candidates inferred from the maximum likelihood (ML) method. The best-fit ML model selection was mtREV24+I [30], and the tree with the highest log likelihood (−21419.90) is shown. The percentage of trees in which the associated taxa clustered together is shown next to the branches. Initial trees for the heuristic search were automatically obtained by applying the Neighbor-joining and BioNJ algorithms to a matrix of pairwise distances estimated using a Jones–Taylor–Thornton (JTT) model and then selecting the topology with the superior log likelihood value. The rate variation model allowed for some sites to be evolutionarily invariable ([+I], 0.00% sites). The tree is drawn to scale, with branch lengths measured in the number of substitutions per site. This analysis included 45 amino acid sequences. All positions with less than 95% site coverage were eliminated, i.e., fewer than 5% alignment gaps, missing data, and ambiguous bases were allowed at any position (partial deletion option). There were 184 positions in the final dataset. Evolutionary analyses were conducted using MEGA X [31].

Previous studies showed that five microbial GTs were validated to have triterpenoid glycosylation activity, including BsYjiC (GenBank Protein accession no. NP_389104) from *B. subtilis* 168 [19,20,21,22,23], UGT109A1 (GenBank Protein accession no. ASY97769) from *B. subtilis* CTCG 63501 [24,28], BsGT1 (GenBank Protein accession no. ANP92054) from *B. subtilis* KCTC 1022 [29], and two GTs, BsUGT398 and BsUGT489, from *B. subtilis* ATCC 6633 (GenBank Protein accession nos. WP_003225398 and WP_003220489, respectively) [4]. To classify which genes were responsible for the biotransformation of GAA, GT genes were first annotated from the GA A07 genome. The 40 identified GT genes were then used to build a phylogenetic tree using the five validated genes with triterpenoid glycosylation activities (Figure 4). Among the 40 GTs, one GT1 (*FQZ25_19010*) and two GT28 (*FQZ25_16345*, *FQZ25_19840*) family genes were most closely related to the five validated genes (marked by stars in Figure 4), and were considered putative gene candidates.

### 2.4. Cloning, Overexpression, and Purification of GT from the GA A07 Strain in E. coli

To obtain the pure GT for the assay of the GAA biotransformation, the three candidate genes (*FQZ25_16345*, *FQZ25_19010*, and *FQZ25_19840*) were subcloned into the pETDuet-1™ expression vector (Figure S1a) and overexpressed with a fusion of His-tag in the amino-terminal in *E. coli* BL21 (DE3), and the produced GT proteins, respectively designated BtGT_16345, BtGT_19010, and BtGT_19840, were purified with Ni^2+^ chelate affinity chromatography. Among them, BtGT_16345 (Figure S1b) and BtGT_19010 (Figure S1d) were successfully purified (shown as a single band in the sodium dodecylsulfate polyacrylamide gel electrophoresis (SDS-PAGE) analysis). In contrast, BtGT_19840 could not be purified due to the insoluble form of the expressed proteins (Figure S1c).

### 2.5. Activity Assays of Recombinant GT Proteins toward GAA

**Figure 5:**
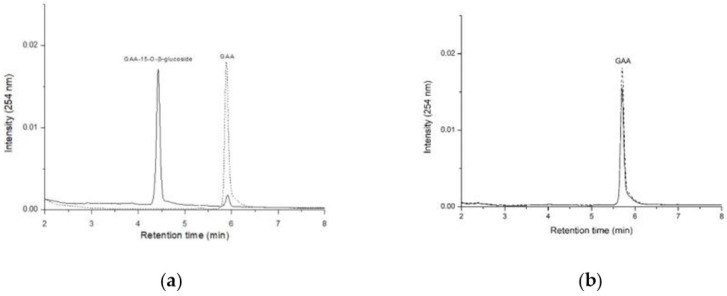
Biotransformation of ganoderic acid A (GAA) by purified BtGT_16345 (**a**) and BtGT_19010 (**b**). Purified enzymes (10 μg/mL) were incubated with 1 mM UDP-glucose and 0.02 mg/mL of GAA in the presence of 50 mM Tris at pH 8.0 and 10 mM MgCl_2_. Before (the 0-min curve) or after (the 30-min curve) 30 min of incubation at 40 °C, the mixtures, together with the standard GAA or GAA-15-*O*-β-glucoside, were analyzed by UPLC. UPLC conditions are described in the Materials and Methods section.

The purified enzymes were incubated with 0.02 mg/mL of GAA, 10 mM of Mg^2+^, and 1 mM of UDP-glucose at pH 8 and 40 °C for 30 min. After incubation, the reaction mixtures were assayed by UPLC. Results showed that BtGT_16345 catalyzed GAA to GAA-15-*O*-β-glucoside (Figure 5a), while BtGT_19010 did not catalyze GAA (Figure 5b).

### 2.6. Catalytic Conditions for BtGT_16345

**Figure 6:**
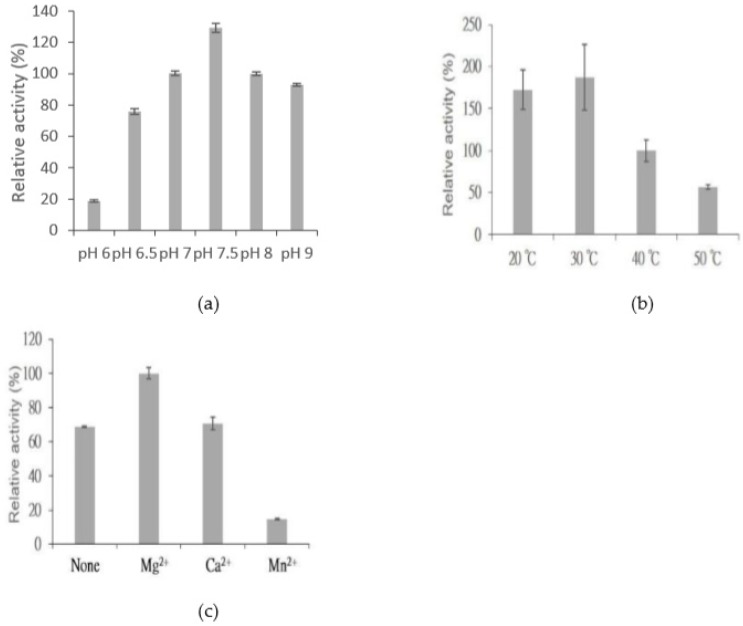
Effects of pH (**a**), temperature (**b**), and metal ions (**c**) on BtGT_16345 activity. The standard condition was set to 10 μg/mL of the purified enzyme, 1 mg/mL of ganoderic acid A (GAA), 10 mM of MgCl_2_, and 10 mM of UDP-glucose in 50 mM of Tris at pH 8.0 and 40 °C. To determine suitable reaction conditions, different pH values, temperatures, and metal ions in the standard condition were replaced with the tested condition. Relative activities were obtained by dividing the area of the product peak of the reaction in the UPLC profile by that of the reaction at the standard condition and are presented as mean values (*n* = 3) along with error bars representing standard deviations.

The activity of purified BtGT_16345 was determined at different pH values and temperatures, and with different metal ions. Many GTs utilize divalent metal ion cofactors, and Mg^2+^ was found to be present in native crystals of some GTs [32]. Results showed that the suitable catalytic conditions for BtGT_16345 protein were at pH 7.5 and 30 °C, with 10 mM of Mg^2+^ (Figure 6).

### 2.7. Substrate Specificity of BtGT_16345

**Figure 7:**
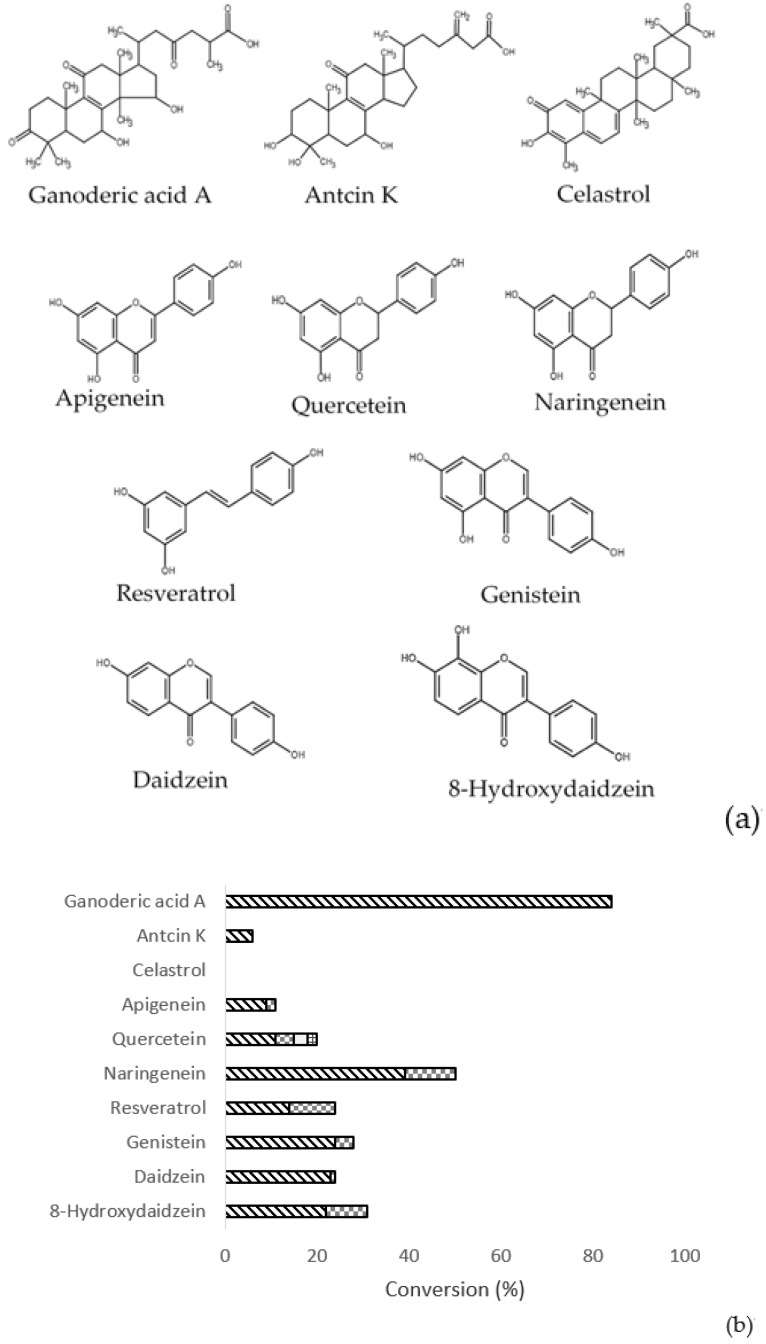
Substrate specificity of BtGT_16345. (**a**) Chemical structures of the test compounds. (**b**) Conversion (%) of the test compounds by BtGT_16345. For the substrate specificity assay, 1 mg/mL of different compounds was mixed with 10 μg/mL of BtGT_16345, 10 mM of UDP-glucose, 10 mM of MgCl_2_, and 50 mM of PB at pH 7.0 and incubated at 30 °C for 30 min. After incubation, the reaction mixture was analyzed by UPLC. UPLC conditions are described in the Materials and Methods section. Conversion of each product was calculated by dividing the peak area of each product by that of the input substrate before biotransformation in the UPLC chromatogram. For biotransformation with multiple products, each product is presented by a bar with a different background pattern.

To determine the substrate specificity of BtGT_16345, the GAA triterpenoid, two additional non-GAA triterpenoids, and seven flavonoids were used as substrates for biotransformation assays (Figure 7a). Conversion of each product was calculated by dividing the peak area of each product by that of the initial input substrate in the UPLC chromatogram. Thus, the calculation of the conversion was only based on the UPLC area due to the different extinctions coefficients of the various products. Our results showed that BtGT_16345 exhibited glycosylation activity toward all tested flavonoids as well as GAA and antcin K, however no activity was detected toward another triterpenoid, celastrol (Figure 7b). In contrast, the purified recombinant BtGT_19010 was not functional on any tested compounds, including GAA.

### 2.8. Kinetic Study of BtGT_16345 toward GAA

**Figure 8:**
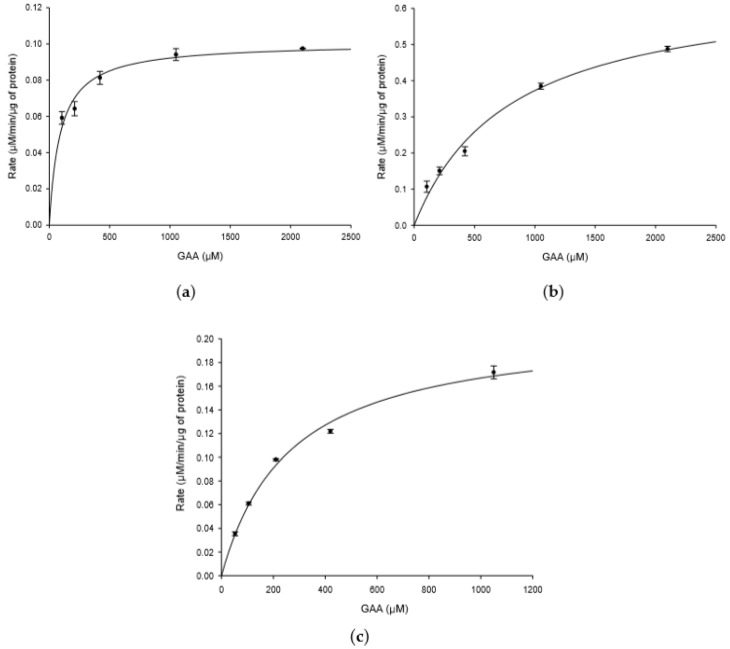
Kinetic study of BsUGT398 (**a**); BsUGT489 (**b**); and BtGT_16345 (**c**). Different concentrations of GAA were mixed with 10 μg purified GT protein, 10 mM UDP-glucose, 10 mM MgCl_2_, and 50 mM PB (pH 7.0) for BtGT_16345, or 50 mM of Tris (pH 8.0) for BsUGT398 and BsUGT489 in 1 mL reaction mixture and incubated at 30 °C for BtGT_16345, or 40 °C for BsUGT398 and BsUGT489 for 20 min. During the incubation, samples from each reaction were removed and analyzed by UPLC every 2 min. The reaction rate for each concentration of GAA was obtained from the slope of the plot of the amount of product over time. The amount of GAA-15-*O*-β-glucoside produced from the reaction was calculated from the peak area of the UPLC analysis normalized to a standard curve. The reaction velocity at each concentration of GAA was obtained from the slope of the plot of time versus the amount of the product and presented as mean values (*n* = 2) along with error bars representing standard deviations. The UPLC operation procedure was described in the Materials and Methods section. The kinetic parameters were calculated by nonlinear regression analysis applied to Michaelis-Menten equation as the description in the Materials and Methods section.

Figure 1 shows that *B. thuringiensis* GA A07 possessed higher GAA biotransformation activity than *B. subtilis* ATCC 6633. Moreover, the GTs of *B. subtilis* ATCC 6633 responsible for catalyzing the biotransformation were identified as BsUGT398 and BsUGT489 in a previous study [4]. To compare the catalytic efficiency of GTs toward GAA from the two strains, a kinetic study was performed on the three GTs: BsUGT398, BsUGT489, and BtGT_16345. Both recombinant purified BsUGT398 and BsUGT489 were obtained from a previous study [4]. The kinetic study was performed by using different concentrations of GAA as the substrate for the individual testing GT enzyme and the reaction velocity at each concentration of GAA was obtained from the slope of the plot of time versus the amount of the product (Figure 8). The kinetic parameters were calculated by nonlinear regression analysis applied to Michaelis–Menten equation (Table 1). Results showed that BsUGT398 exhibited the significant highest GAA-binding affinity with a K_M_ value of 90.71 ± 14.86 µM, while BsUGT489 exhibited the highest turnover number with a k_cat_ value of 0.9336 ± 0.0626 s^−1^. However, the catalytic efficiency (k_cat_/K_M_) of BtGT_16345 did not show significantly different compared with either BsUGT398 or BsUGT489 (Table 1).

## 3. Discussion

This study sequenced and assembled the complete genome of the GA A07 strain for strain classification and GT identification. The circular map in Figure 2 not only contained critical information about the genome (including the numbers of protein-coding genes, ribosomal RNA genes, and tRNA genes, and the GC content distribution on the genome) but also revealed several interesting characteristics. For example, the GC proportions were higher on the rRNA regions, and that the rRNA genes were grouped in a portion of the genome (especially between positions 1.2 M to 1.5 M) instead of distributed evenly on the genome. The distribution of the protein-coding genes were also uneven for both forward and reverse-complement strands, in which genes in forward strand were more abundant in half of the genome (from positions 1.2 M to 3.75 M) while genes in reverse-complement strand were in greater number in another half of the genome. We consider these characteristics outside the scope of this manuscript but may warrant future genome analysis to find their underlying meanings.

The phylogenetic tree built from 16S genes could only identify that the phylogenetic placement of the GA A07 strain was very closely related to a group of *Bacilli*, including *B. thuringiensis*, *B. anthracis*, *B. cereus*, and *B. subtilis* [18]. Herein, a three-step approach was applied to identify strain GA A07: (1) downloading all available *Bacillus* genomes; (2) applying ANIs, AAIs, and a TCS to pinpoint the most closely related species/strain among *Bacillus*; and (3) building a phylogenetic tree using 250 single-copy marker genes. This approach helped us reclassify strain GA A07 as a part of *B. thuringiensis* (Figure 3). Figure 4 further shows putative GTs identified from the novel genome sequence and three GT candidates, one GT1 (BtGT_19010) and two GT28s (BtGT_16345, BtGT_19840), were grouped with the five triterpenoid-glycosylation GTs (BsYjiC, UGT109A1, BsGT1, BsUGT398, and BsUGT489).

Previous studies identified *B. subtilis* ATCC 6633 and *B. thuringiensis* GA A07 were able to biotransform GAA into GAA-15-*O*-β-glucoside [4,18]. Herein *B. thuringiensis* GA A07 exhibited over 10-fold higher biotransformation activity than *B. subtilis* ATCC 6633 (Figure 1). Through a genome-centric analysis, we further identified that BtGT_16345, which belongs to the GT28 family, exhibited good glycosylation activity toward GAA (Figure 5). This is the first report that a GT28 enzyme, not only GT1, can catalyze GAA triterpenoids. From the results of the kinetics study, the catalytic efficiency (k_cat_/K_M_) of BtGT_16345 for *B. thuringiensis* GA A07 did not show significantly different compared with either BsUGT398 or BsUGT489 for *B. subtilis* ATCC 6633 (Table 1). Therefore, BtGT_16345 identified in *B. thuringiensis* GA A07 might not be the major contributor to 10-fold higher biotransformation activity. BtGT_19840, the activity of which could not be evaluated in this study owing to an inability to obtain its soluble expression, may contribute to the higher GAA biotransformation of *B. thuringiensis*. Other possible reasons include: (1) higher expression of BtGT with GAA biotransformation activity, (2) a higher uptake rate of GAA into cells; (3) a higher UDP-glucose concentration accumulating in cells, and (4) other enzymes and/or coenzymes cooperating in cells, may account for the contribution of the higher catalytic efficiency of *B. thuringiensis* GA A07.

Although over 500,000 GTs were identified in the CAZy database, only six microbial GTs were found to exhibit glycosylation activities toward triterpenoids, including BtGT_16345 (this study), BsYjiC [19,20,21,22,23], UGT109A1 [24,28], BsGT1 [29], BsUGT398, and BsUGT489 [4]. BtGT_16345, which was classified into the GT28 family, is the only exception out of the other five GTs that belong to the GT1 family [1]. Previous studies showed that these GT1 enzymes catalyzed the glycosylation of several flavonoids at multiple positions to form mixture of flavonoid glycosides [19,20,21,22,23,24,28,29]. In the present study, BtGT_16345 exhibited similar catalytic activities toward seven flavonoids to form multiple products (Figure 7). These results revealed that microbial GTs potentially accept a broader range of different flavonoids as catalytic substrates and exhibit less regioselectivity toward flavonoid substrates than do plant GTs [2,3].

As to triterpenoid substrates, both BsYjiC [19,20,21,22,23] and UGT109A1 [24,28] catalyzed *O*-glycosylation toward triterpenoids at multiple positions (C-3, C-6, C-12, and C-20). As a result, one triterpenoid substrate can potentially be biotransformed into many types of triterpenoid glycosides. The above reports revealed that the two GT1s were less regioselective toward triterpenoid substrates. In contrast, BtGT_16345 specifically catalyzed glycosylation at the C-15 position out of the three hydroxyl groups capable of the *O*-glycosylation (C-7, C-15, and C-26) of GAA and no other glycosylated products were detected during the biotransformation using the analytical techniques herein (Figure 5). Moreover, only one product was produced by BtGT_16345 from the biotransformation toward antcin K, another triterpenoid containing four sites capable of *O*-glycosylation (C-3, C-4, C-7, and C-26) (Figure 7). BtGT_16345 was regioselective toward triterpenoid substrates, and could be further used for industrial applications or stepwise biosynthesis for structure-activity studies.

## 4. Materials and Methods

### 4.1. Microorganism and Chemicals

The *Bacillus* sp. GA A07 strain was isolated from intestinal bacteria of zebrafish in our previous study [18]. Both purified recombinant BsUGT398 and BsUGT489 were obtained in our previous study [4]. Antcin K was obtained by a procedure in our previous study [33]. GAA and celastrol were bought from Baoji Herbest Bio-Tech (Xi-An, Shaanxi, China). 8-OHDe was prepared according to Wu et al.’s [34] method. Other flavonoids were purchased from Sigma (St. Louis, MO, USA) or Tokyo Chemical Industry (Tokyo, Japan). UDP-glucose was purchased from Cayman Chemical (Ann Arbor, MI, USA). All materials needed for the polymerase chain reaction (PCR), including primers, deoxyribonucleotide triphosphate, and Taq DNA polymerase, were purchased from MDBio (Taipei, Taiwan). The pETDuet-1 plasmid was purchased from Novagen (Madison, WI, USA). Restriction enzymes and DNA ligase were obtained from New England Biolabs (Ipswich, MA, USA). Other reagents and solvents used were of high quality, and were purchased from commercially available sources.

### 4.2. Whole-Genome Sequencing

*Bacillus* sp. GA A07 cells were harvested from Luria-Bertani (LB) plates pre-cultured in a 25 °C incubator for 3 days. Genomic DNA of cells was extracted with a ZR Soil Microbe DNA Kit™ (D6001, Zymo Research, Irvine, CA, USA). Genomic DNA (1~3 μg) was end-repaired and ligated with NB12 barcode sequencing adaptors (EXP-NBD104, Native Barcoding Expansion 1-12) via a KAPA Hyper Prep Kit (Cat#KR0961, Kapa Biosystems, Wilmington, MA, USA), following the manufacturer’s instructions. The barcoded genomic DNA library was premixed with LB and SQB buffer of the Ligation Sequencing Kit (SQK-LSK109, Oxford Nanopore Technologies, UK) loaded in a Flow Cell (R9.4.1; FLO-MIN106), and sequenced by MinION devices for 24 h. For short-read sequencing, the genomic DNA library was fragmented by Covaris S220 with a 350-bp size peak, a peak incident power of 140 W, a duty factor of 10, 200 cycles per burst, and a treatment time of 100 s. Sheared DNA fragments were then used for library construction with the MGIEasy DNA Library Prep Kit v1.1. The library was sequenced with the BGISEQ-500RS sequencer by Tri-I Biotech (New Taipei, Taiwan).

### 4.3. Genome Assembly and Annotation

Nanopore reads were first error-corrected using canu v1.8 [35] (parameters: -correct -nanopore-raw); the error-corrected reads were then assembled using wtdbg2 v2.4 [36]. The yielded assembly was mapped to BGISEQ-500RS paired-end sequences (trimmed using SOAPnuke v2.0.7 [37]; parameters: filter -l 10 -q 0.5 -n 0.1 -M 2 --adaMR 0.5 -f AAGTCGGAGGCCAAGCGGTCTTAGGAAGACAA -r AAGTCGGATCGTAGCCATGTCGTTCTGTGAGCCAAGGAGTTG) using bowtie2v 2.2.3 [38]. The mapping SAM file was first converted to a sorted indexed BAM file using SAMtools v1.9-45 [39] (samtools sort; samtools index) and then used as input for the assembly error correction tool, Pilon v1.23 [40].

### 4.4. Reclassification of GA A07 Strain

In total, 882 *Bacillus* genomes were downloaded to identify the most closely related species. FastANI v1.2 [41] was leveraged to identify the most closely related species (i.e., the ones with the highest average nucleotide identity compared to the GA A07 genome). Average amino acid identity was also checked by the following steps: (1) Prodigal v2.6.3 [25] was used to in batch-predict protein-coding genes from the genomes, and genes were converted into amino acid sequences; (2) amino acid identities between the GA A07 genome and all downloaded *Bacillus* genomes were compared using BLASTP [26] (parameters: -max_target_seqs 1 -evalue 1e-10); and (3) the mean value of the best-hit identities was calculated and was regarded as the average amino acid identity between the two genomes. The Tetra Correlation Search (TCS) function implemented in the JSpeciesWS webserver [27] was also pursued to find the most closely related bacterial genome based on tetra-nucleotide composition evidence.

Only genomes with better assembly quality (defined as genomes with at most ten scaffolds) were used to build the phylogenetic tree. The tree was generated using ezTree [42], which is capable of identifying single-copy marker genes among input genomes, thereby creating a concatenated alignment of all marker genes, and using FastTree 2 [43] with the Jones–Taylor–Thornton (JTT) evolutionary model and 1000 resampling tests to construct a reliable tree. The nwk file of the phylogenetic tree was then visualized using Molecular Evolutionary Genetics Analysis (MEGA) X software [31].

### 4.5. Identification and Analysis of GT Genes

The dbCAN2 webserver [44] was employed to identify potential GTs from the *B. thuringiensis* GA A07 genome. An unrooted phylogenetic tree of all extracted GT protein sequences was constructed using MEGA X software [31] with the maximum-likelihood method, 500 bootstrap replications, the general reversible mitochondrial model [30], and partial deletion.

### 4.6. Fermentation and Biotransformation of GAA

*Bacillus subtilis* ATCC 6633 or *B. thuringiensis* GA A07 was cultivated in a 250-mL baffled Erlenmeyer flask containing 20 mL of LB medium with 5% of glucose at 180 rpm and 28 °C. When the OD_600_ of the cell culture reached 0.6, 1 mg/mL of GAA was added to the broth. Cultivation was carried out for another 32 h, and fermentation broth (0.5 mL) of the culture was taken at predicted time intervals and used for the UPLC analysis to measure the biotransformation activity.

### 4.7. UPLC Analysis

The UPLC system (Acquity UPLC H-Class, Waters, Milford, MA, USA) was equipped with an analytic C18 reversed-phase column (Kinetex^®^ C18, 1.7 µm, 2.1 i.d. × 100 mm, Phenomenex, Torrance, CA, USA). The operating conditions of UPLC for analysis of GAA, antcin K, and celastrol were consistent with those of our previous study [4] except for the 430-nm absorbance detection of celastrol. Operating conditions for flavonoids were from our previous study [11].

### 4.8. Expression and Purification of GT from GA A07 Strain

Genomic DNA of the GA A07 strain was isolated using the commercial kit Geno *Plus*^TM^ (Viogene, Taipei, Taiwan). Candidate GT genes were amplified from genomic DNA using a PCR with specific primer sets (Table S2). The amplified GT genes were subcloned into the pETDuet-1™ vector through suitable restriction enzyme sites (Table S2) to obtain the expression vector, pETDuet-BtGT (Figure S1a). Expression vectors were transformed into *E. coli* BL21 (DE3) via electroporation to obtain recombinant *E. coli*.

Recombinant BtGT_16345, BtGT_19840, and BtGT_19010 were produced and purified from the recombinant *E. coli*, and analyzed by SDS-PAGE (Figure S1b–d). The protein concentration was determined by a Bradford assay using bovine serum albumin as the standard. The experimental procedures were the same as those in our previous study [4].

### 4.9. In Vitro Biotransformation Assay

In vitro biotransformation was performed using purified GT proteins. In a 0.1mL standard reaction mixture, 1 μg of purified GT protein, 0.02 mg/mL of GAA, 1 mM of UDP-glucose, 10 mM of MgCl_2_, and 50 mM of Tris at pH 8.0 were added. The reaction was carried out at 40 °C for 30 min, stopped by adding 0.9 mL of methanol, and analyzed by UPLC.

For optimization experiments, different pH values, temperatures, and metal ions were replaced in the standard reaction, where 1 mg/mL of GAA was used. For pH testing, PB at pH 6.0 to 7.5, and Tris buffer at pH 8.0 and pH 9.0 were used. For metal ion testing, 10 mM of MgCl_2_, CaCl_2_, or MnCl_2_ was used. The relative activity was obtained by dividing the area of the product peak of the reaction in the UPLC profile by that of the reaction with Tris pH 8.0, at 40 °C, and with 10 mM of MgCl_2_.

For the substrate specificity assay, 25 mg/mL of the substrate soluble in dimethyl sulfoxide conditions, all tested substances were soluble in the reaction buffer. 1 mg/mL of different test compounds was mixed with 1 μg of purified GT protein, 10 mM of UDP-glucose, 10 mM of MgCl_2_, and 50 mM of PB pH 7.0 in a 0.1mL reaction mixture and incubated at 30 °C for 30 min. After incubation, the reaction mixture was analyzed by UPLC.

For the kinetic experiments, different concentrations of GAA were mixed with 10 μg of purified GT protein, 10 mM of UDP-glucose, 10 mM of MgCl_2_, and 50 mM of PB at pH 7.0 for BtGT_16345, or 50 mM of Tris at pH 8.0 for BsUGT398 and BsUGT489 in a 1-mL reaction mixture and incubated at 30 °C for BtGT_16345, or 40 °C for BsUGT398 and BsUGT489 for 20 min. During incubation, samples from each reaction were taken out and analyzed by UPLC every 2 min. The amount of GAA-15-*O*-β-glucoside produced from the reaction was calculated from the peak area of the UPLC analysis normalized to a standard curve. The reaction velocity at each concentration of GAA was obtained from the slope of the plot of time versus the amount of the product. The kinetic parameters were calculated by nonlinear regression analysis applied to Michaelis-Menten equation using SigmaPlot 14.0 software (Systat Software, San Jose, CA, USA). The k_cat_ values were calculated using the predicted molecular mass for each recombinant enzyme.

## 5. Conclusions

A novel GT28 family enzyme, BtGT_16345, from a new genome assembly of the *B. thuringiensis* GA A07 strain, was identified that can biotransform GAA into GAA-15-*O*-β-glucoside. To our knowledge, BtGT_16345 is the first GT28 family enzyme with triterpenoid glycosylation activity.

## Figures and Tables

**Table 1:** Kinetic parameters of GT toward GAA.

GT	K_M_ (μM)	k_cat_ (s^−1^)	k_cat_/K_M_ (s^−1^ mM^−1^)
BsUGT398	90.71 ± 14.86	0.1401 ± 0.0051	1.5445 ± 0.2592
BsUGT489	793.96 ± 124.09	0.9336 ± 0.0626	1.1759 ± 0.2000
BtGT_16345	263.82 ± 24.78	0.2944 ± 0.0109	1.1159 ± 0.1127

## Supplementary Materials

Supplementary materials can be found at https://www.mdpi.com/1422-0067/20/20/5192/s1. The following materials are available online. Table S1: Single copy marker genes used for building the phylogenetic tree; Table S2: Nucleotide sequences of the primers used for amplification of glycosyltransferase (GT) genes in the present study; Figure S1: Expression and purification of glycosyltransferases (GTs) from *Bacillus thuringiensis* GA A07 in *E. coli*.

## Abbreviations

GAA	Ganoderic acid A
GT	Glycosyltransferase
UDP	Uridine diphosphate
