# Supplementary material for: A Genome-Centric Approach Reveals a Novel Glycosyltransferase from the GA A07 Strain of Bacillus thuringiensis Responsible for Catalyzing 15-O-Glycosylation of Ganoderic Acid A
Source: Int J Mol Sci. 2019 Oct 20;20(20):5192. doi: 10.3390/ijms20205192 (PMC6829469; doi:10.3390/ijms20205192)
Supplement: Supplementary file 1 [file ijms-20-05192-s001.pdf]

A Genome-Centric Approach Reveals a Novel Glycosyltransferase from the GA A07 Strain of *Bacillus thuringiensis* Responsible for Catalyzing 15-*O*-Glycosylation of Ganoderic Acid A

Te-Sheng Chang [1,†], Tzi-Yuan Wang [2,†], Tzu-Yu Hsueh [1], Yu-Wen Lee [1], Hsin-Mei Chuang [1], Wen-Xuan Cai [1], Jiumn-Yih Wu [3], Chien-Min Chiang [4] and Yu-Wei Wu [5,6,*]

Table S1. Single copy marker genes used for building the phylogenetic tree.

Pfam ID	Pfam description
PF03775.15	MinC_C, Septum
PF01406.18	tRNA-synt_1e, tRNA
PF00016.19	RuBisCO_large, Ribulose
PF00366.19	Ribosomal_S17, Ribosomal
PF01071.18	GARS_A, Phosphoribosylglycinamide
PF01729.18	QRPTase_C, Quinolinate
PF00177.20	Ribosomal_S7, Ribosomal
PF06135.11	DUF965, Bacterial
PF00380.18	Ribosomal_S9, Ribosomal
PF13662.5	Toprim_4, Toprim
PF07307.10	HEPPP_synt_1, Heptaprenyl
PF03947.17	Ribosomal_L2_C, Ribosomal
PF01933.17	UPF0052, Uncharacterised
PF00420.23	Oxidored_q2, NADH-ubiquinone/plastoquinone
PF12631.6	MnmE_helical, MnmE
PF00416.21	Ribosomal_S13, Ribosomal
PF00327.19	Ribosomal_L30, Ribosomal
PF02504.14	FA_synthesis, Fatty
PF00297.21	Ribosomal_L3, Ribosomal
PF01219.18	DAGK_prokar, Prokaryotic
PF06838.10	Met_gamma_lyase, Methionine
PF14437.5	MafB19-deam, MafB19-like
PF04296.12	DUF448, Protein
PF01676.17	Metalloenzyme, Metalloenzyme
PF00430.17	ATP-synt_B, ATP
PF01152.20	Bac_globin, Bacterial-like
PF01964.17	ThiC_Rad_SAM, Radical
PF08676.10	MutL_C, MutL
PF00885.18	DMRL_synthase, 6,7-dimethyl-8-ribityllumazine
PF02152.17	FolB, Dihydroneopterin
PF00453.17	Ribosomal_L20, Ribosomal
PF01245.19	Ribosomal_L19, Ribosomal
PF13331.5	DUF4093, Domain
PF00406.21	ADK, Adenylate
PF02261.15	Asp_decarbox, Aspartate
PF08529.10	NusA_N, NusA
PF12344.7	UvrB, Ultra-violet
PF01409.19	tRNA-synt_2d, tRNA
PF01208.16	URO-D, Uroporphyrinogen

PF09382.9	RQC, RQC
PF00444.17	Ribosomal_L36, Ribosomal
PF14450.5	FtsA, Cell
PF01592.15	NifU_N, NifU-like
PF00347.22	Ribosomal_L6, Ribosomal
PF00290.19	Trp_syntA, Tryptophan
PF08323.10	Glyco_transf_5, Starch
PF01127.21	Sdh_cyt, Succinate
PF17136.3	ribosomal_L24, Ribosomal
PF02699.14	YajC, Preprotein
PF12002.7	MgsA_C, MgsA
PF04445.12	SAM_MT, Putative
PF00318.19	Ribosomal_S2, Ribosomal
PF00121.17	TIM, Triosephosphate
PF06265.10	DUF1027, Protein
PF04297.13	UPF0122, Putative
PF02130.16	UPF0054, Uncharacterized
PF00218.20	IGPS, Indole-3-glycerol
PF02390.16	Methyltransf_4, Putative
PF03948.13	Ribosomal_L9_C, Ribosomal
PF02540.16	NAD_synthase, NAD
PF02410.14	RsfS, Ribosomal
PF01649.17	Ribosomal_S20p, Ribosomal
PF00164.24	Ribosom_S12_S23, Ribosomal
PF00673.20	Ribosomal_L5_C, ribosomal
PF00237.18	Ribosomal_L22, Ribosomal
PF00189.19	Ribosomal_S3_C, Ribosomal
PF05698.13	Trigger_C, Bacterial
PF02576.16	DUF150, RimP
PF01106.16	NifU, NifU-like
PF00163.18	Ribosomal_S4, Ribosomal
PF00800.17	PDT, Prephenate
PF04452.13	Methyltrans_RNA, RNA
PF02631.15	RecX, RecX
PF02578.14	Cu-oxidase_4, Multi-copper
PF00203.20	Ribosomal_S19, Ribosomal
PF06962.11	rRNA_methylase, Putative
PF06569.10	DUF1128, Protein
PF02620.16	DUF177, Uncharacterized
PF00886.18	Ribosomal_S16, Ribosomal
PF04472.11	SepF, Cell
PF13025.5	DUF3886, Protein
PF09924.8	DUF2156, Uncharacterised
PF00687.20	Ribosomal_L1, Ribosomal
PF06335.11	DUF1054, Protein
PF13509.5	S1_2, S1
PF00252.17	Ribosomal_L16, Ribosomal
PF13684.5	Dak1_2, Dihydroxyacetone
PF02618.15	YceG, YceG-like
PF07435.10	YycH, YycH
PF13275.5	S4_2, S4
PF02153.16	PDH, Prephenate

PF03205.13	MobB, Molybdopterin
PF00828.18	Ribosomal_L27A, Ribosomal
PF00137.20	ATP-synt_C, ATP
PF01250.16	Ribosomal_S6, Ribosomal
PF02616.13	SMC_ScpA, Segregation
PF05991.10	NYN_YacP, YacP-like
PF07475.11	Hpr_kinase_C, HPr
PF00709.20	Adenylsucc_synt, Adenylosuccinate
PF00312.21	Ribosomal_S15, Ribosomal
PF02666.14	PS_Dcarbxylase, Phosphatidylserine
PF01746.20	tRNA_m1G_MT, tRNA
PF02565.14	RecO_C, Recombination
PF02142.21	MGS, MGS-like
PF13507.5	GATase_5, CobB/CobQ-like
PF08534.9	Redoxin, Redoxin
PF02882.18	THF_DHG_CYH_C, Tetrahydrofolate
PF01782.17	RimM, RimM
PF03946.13	Ribosomal_L11_N, Ribosomal
PF02367.16	TsaE, Threonylcarbamoyl
PF02545.13	Maf, Maf-like
PF09182.9	PuR_N, Bacterial
PF01865.15	PhoU_div, Protein
PF00490.20	ALAD, Delta-aminolevulinic
PF04468.11	PSP1, PSP1
PF02033.17	RBFA, Ribosome-binding
PF03602.14	Cons_hypoth95, Conserved
PF00707.21	IF3_C, Translation
PF01196.18	Ribosomal_L17, Ribosomal
PF01288.19	HPPK, 7,8-dihydro-6-hydroxymethylpterin-pyrophosphokinase
PF04997.11	RNA_pol_Rpb1_1, RNA
PF00825.17	Ribonuclease_P, Ribonuclease
PF10635.8	DisA-linker, DisA
PF01379.19	Porphobil_deam, Porphobilinogen
PF04260.11	DUF436, Protein
PF04263.15	TPK_catalytic, Thiamin
PF02401.17	LYTB, LytB
PF00542.18	Ribosomal_L12, Ribosomal
PF00773.18	RNB, RNB
PF06415.12	iPGM_N, BPG-independent
PF01509.17	TruB_N, TruB
PF00573.21	Ribosomal_L4, Ribosomal
PF01192.21	RNA_pol_Rpb6, RNA
PF01634.17	HisG, ATP
PF04816.11	TrmK, tRNA
PF01252.17	Peptidase_A8, Signal
PF00926.18	DHBP_synthase, 3,4-dihydroxy-2-butanone
PF08756.9	YfkB, YfkB-like
PF01502.17	PRA-CH, Phosphoribosyl-AMP
PF13797.5	Post_transc_reg, Post-transcriptional
PF01668.17	SmpB, SmpB
PF11009.7	DUF2847, Protein
PF12072.7	DUF3552, Domain

PF14116.5	YyzF, YyzF-like
PF06156.12	YabB, Initiation
PF00308.17	Bac_DnaA, Bacterial
PF02445.15	NadA, Quinolinate
PF04551.13	GcpE, GcpE
PF14489.5	QueF, QueF-like
PF01205.18	UPF0029, Uncharacterized
PF00276.19	Ribosomal_L23, Ribosomal
PF01632.18	Ribosomal_L35p, Ribosomal
PF09648.9	YycI, YycH
PF00584.19	SecE, SecE/Sec61-gamma
PF02934.14	GatB_N, GatB/GatE
PF01325.18	Fe_dep_repress, Iron
PF04238.11	DUF420, Protein
PF13085.5	Fer2_3, 2Fe-2S
PF01628.20	HrcA, HrcA
PF04025.11	DUF370, Domain
PF01165.19	Ribosomal_S21, Ribosomal
PF00238.18	Ribosomal_L14, Ribosomal
PF02542.15	YgbB, YgbB
PF00889.18	EF_TS, Elongation
PF00118.23	Cpn60_TCP1, TCP-1/cpn60
PF08275.10	Toprim_N, DNA
PF00166.20	Cpn10, Chaperonin
PF02464.16	CinA, Competence-damaged
PF00815.19	Histidinol_dh, Histidinol
PF02609.15	Exonuc_VII_S, Exonuclease
PF01121.19	CoaE, Dephospho-CoA
PF01884.16	PcrB, PcrB
PF02773.15	S-AdoMet_synt_C, S-adenosylmethionine
PF02700.13	PurS, Phosphoribosylformylglycinamidine
PF14681.5	UPRTase, Uracil
PF02580.15	Tyr_Deacylase, D-Tyr-tRNA(Tyr)
PF08741.9	YwhD, YwhD
PF01795.18	Methyltransf_5, MraW
PF05746.14	DALR_1, DALR
PF00411.18	Ribosomal_S11, Ribosomal
PF02547.14	Queuosine_synth, Queuosine
PF07136.10	DUF1385, Protein
PF02569.14	Pantoate_ligase, Pantoate-beta-alanine
PF16199.4	Radical_SAM_C, Radical_SAM
PF03840.13	SecG, Preprotein
PF07187.10	DUF1405, Protein
PF08442.9	ATP-grasp_2, ATP-grasp
PF03079.13	ARD, ARD/ARD'
PF03118.14	RNA_pol_A_CTD, Bacterial
PF00466.19	Ribosomal_L10, Ribosomal
PF00410.18	Ribosomal_S8, Ribosomal
PF02325.16	YGGT, YGGT
PF07497.11	Rho_RNA_bind, Rho
PF00861.21	Ribosomal_L18p, Ribosomal
PF01715.16	IPPT, IPP

PF08459.10	UvrC_HhH_N, UvrC
PF01195.18	Pept_tRNA_hydro, Peptidyl-tRNA
PF02628.14	COX15-CtaA, Cytochrome
PF01171.19	ATP_bind_3, PP-loop
PF00809.21	Pterin_bind, Pterin
PF00231.18	ATP-synt, ATP
PF01678.18	DAP_epimerase, Diaminopimelate
PF02590.16	SPOUT_MTase, Predicted
PF01176.18	eIF-1a, Translation
PF05848.10	CtsR, Firmicute
PF06257.10	VEG, Biofilm
PF00217.18	ATP-gua_Ptrans, ATP:guanido
PF01783.22	Ribosomal_L32p, Ribosomal
PF06418.13	CTP_synth_N, CTP
PF01259.17	SAICAR_synt, SAICAR
PF00850.18	Hist_deacetyl, Histone
PF03477.15	ATP-cone, ATP
PF00830.18	Ribosomal_L28, Ribosomal
PF01084.19	Ribosomal_S18, Ribosomal
PF13277.5	YmdB, YmdB-like
PF00227.25	Proteasome, Proteasome
PF03483.16	B3_4, B3/4
PF13380.5	CoA_binding_2, CoA
PF01330.20	RuvA_N, RuvA
PF10437.8	Lip_prot_lig_C, Bacterial
PF03652.14	RuvX, Holliday
PF14527.5	LAGLIDADG_WhiA, WhiA
PF00636.25	Ribonuclease_3, Ribonuclease
PF07408.10	DUF1507, Protein
PF10141.8	ssDNA-exonuc_C, Single-strand
PF00475.17	IGPD, Imidazoleglycerol-phosphate
PF01761.19	DHQ_synthase, 3-dehydroquinate
PF01765.18	RRF, Ribosome
PF13047.5	DUF3907, Protein
PF00562.27	RNA_pol_Rpb2_6, RNA
PF00831.22	Ribosomal_L29, Ribosomal
PF02686.14	Glu-tRNAGln, Glu-tRNAGln
PF00338.21	Ribosomal_S10, Ribosomal
PF06778.11	Chlor_dismutase, Chlorite
PF00334.18	NDK, Nucleoside
PF03668.14	ATP_bind_2, P-loop
PF02224.17	Cytidylate_kin, Cytidylate
PF10369.8	ALS_ss_C, Small
PF10418.8	DHODB_Fe-S_bind, Iron-sulfur
PF02568.13	ThiI, Thiamine
PF04079.15	SMC_ScpB, Segregation
PF12836.6	HHH_3, Helix-hairpin-helix
PF03309.13	Pan_kinase, Type
PF03772.15	Competence, Competence
PF01070.17	FMN_dh, FMN-dependent
PF06071.12	YchF-GTPase_C, Protein
PF00213.17	OSCP, ATP

PF00677.16	Lum_binding, Lumazine
PF02575.15	YbaB_DNA_bd, YbaB/EbfC
PF00694.18	Aconitase_C, Aconitase

Table S2. Nucleotide sequences of the primers used for amplifications of GT genes in the present study.

Name	Nucleotide sequence of the primer [1]
BtGT_16345F-BamHI	CCCGGATCCGGCAAATGTACTCGTAATAAATTTCC
BtGT_16345R-XhoI	CCCCTCGAGCTATTTAATCTTTACGTACGGCTTC
BtGT_19840F-EcoRI	CCCGAATTCGGCGCGTGTTTTATTTATTAATGC
BtGT_19840R-XhoI	CCCCTCGAGTCACTGACCTACAAATATAAAAATTTC
BtGT_19010F-EcoRI	CCCGAATTCGCTAAATATTTTAGTAGTTAATTTTCC
BtGT_19010R-XhoI	CCCCTCGAGTTAAAGTTTTACTTTATTTAAAAGAG

[1] The sequences for the designed restriction enzyme sites are noted by underline.

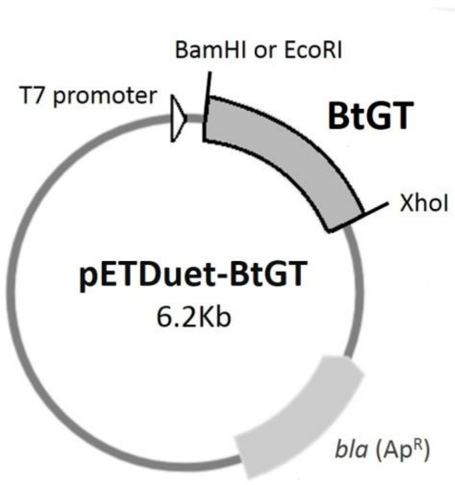

(**a**)

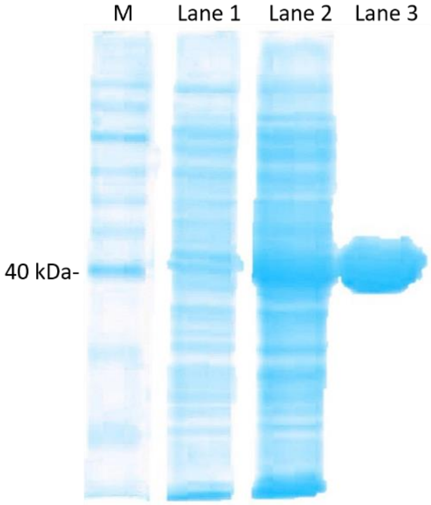

(**b**)

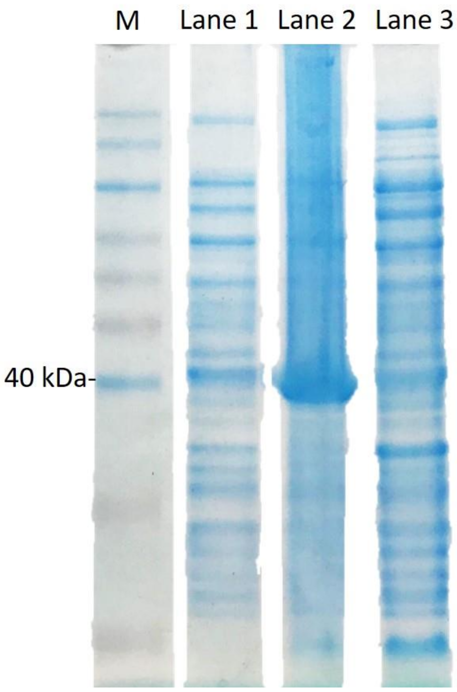

(**c**)

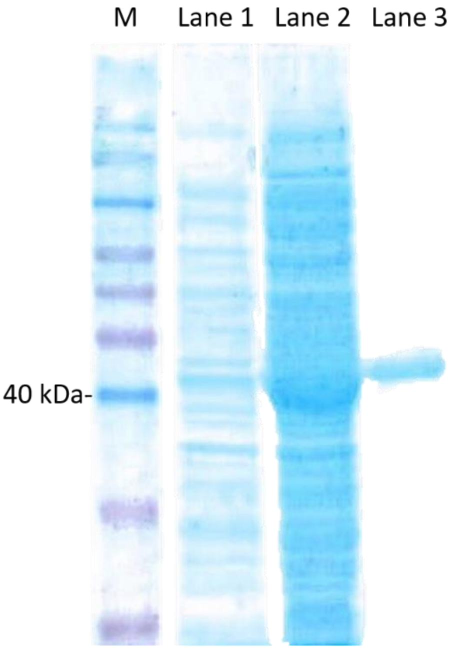

(**d**)

Figure S1. Expression and purification of GT from *B. thuringiensis* GA A07 in *E. coli*. Diagram of the recombinant repression plasmid (a). SDS-PAGE analysis of expressed and purified proteins from recombinant *E. coli* harboring pETDuet-BtGT_16345 (**b**), pETDuet-BtGT_19840 (**c**) or pETDuet-BtGT_19010 (**d**). Lane M: molecular marker; lane 1: total protein before induction; lane 2: total protein after 20 h induction; lane 3 in (**b**) and (**d**): purified protein; in (**c**): total protein of 20 h induction after centrifugation to remove insoluble proteins.
